# Effect of pollen and resource limitation on reproduction of *Zygophyllum xanthoxylum* in fragmented habitats

**DOI:** 10.1002/ece3.3465

**Published:** 2017-09-26

**Authors:** Min Chen, Xue‐Yong Zhao

**Affiliations:** ^1^ Northwest Institute of Eco‐Environment and Resources CAS Lanzhou China

**Keywords:** pollen limitation, pollinator behavior, pollinators, resource limitation, seed set

## Abstract

Limitations on pollen and resources may significantly affect plant reproduction in fragmented habitats. In this study, phenology and pollinator frequency and activity were investigated to estimate the role of pollinators in *Zygophyllum xanthoxylum* reproduction, and this species is ecologically important in northwest China. In addition, the relative impact of restrictive amounts of pollen and resources on the seed set per flower was evaluated. It was found that adding pollen boosted the size of the seed set per flower, but had no significant effect on the number of flowers. By contrast, the addition of resources increased flower numbers as well as had a slight impact on the seed set per flower. These results indicate the amount of available pollen is a limiting factor for reproductive success. Moreover, *Apis mellifera* was identified as the most effective pollinator of *Z. xanthoxylum*, and there were more overall pollinators and visitations in the control than in the fragmented habitats. Furthermore, the limitations in pollen were more restrictive in the fragmented area than in the control. This was due to increased pollinator visitations in the control that could ameliorate the effects of lower pollen levels. When there is a limited availability of suitable pollinators, self‐pollination is critical in fragmented habitats. *Z. xanthoxylum* has reproductive strategies that aid in adapting to harsh environments, including protogyny and delayed selfing.

## 1 INTRODUCTION

The sex allocation model has highly influenced the field of plant reproductive ecology (Goldman & Willson, 1986). This model is based on the observation that a significant proportion of flowers and ovules in a plant do not become fruit or seeds (Lloyd, 1980; Stephenson, 1981). Several hypotheses have been put forth concerning this phenomenon, including restriction of pollen and resources, that is, pollen limitation (Burd, 1994; Sutherland, 1987). Pollen limitation refers to pollen quantities or quality that hinders the reproductive success of plants (Aizen & Harder, 2007; Ashman et al., 2004). The most frequently studied example of this is insufficient pollinator service, which especially has an effect on selection for floral traits relating to the pollinator activity in animal‐pollinated plants (Burd, 1994; Liao, Song, & Zhang, 2006; Suzuki, 2000; Yang, Sun, & Guo, 2005). In addition, the distance between plants can affect a number of pollination‐related processes, such as attracting pollinators and breeding systems (Ashman & Morgan, 2004; Kearns, Inouye, & Waser, 1998). Restriction of pollen and resources is often encountered by plants influenced by natural pollination conditions and may lead to low reproductive output (Asikainen & Mutikainen, 2005; Knight et al., 2005). Many studies have suggested that it should be assumed plants are limited by resource availability if the reproductive output of an individual plant does not increase after supplemental hand pollination (Whigham, 1984; Zimmerman & Aide, 1989).

Human impacts on landscapes usually result in habitat fragmentation and disrupt pollination process (Hobbs & Yates, 2003; Saunders, Hobbs, & Margules, 1991). Habitat fragmentation results in isolation effect and creates conditions that adversely affect the survival of many species, thereby disrupting species interactions such as plant–animal mutualisms (Kolb, 2005; Rodríguez‐Cabal, Aizen, & Novaro, 2007). Fragmented habitats can change the foraging patterns of pollinators, affect pollinator behavior, and even affect the pollination success of plants and plant fitness (Cresswell, 1997; Dyck & Matthysen, 1999). If the distance between plants to be pollinated is too large, pollination is limited and plant fitness may be reduced due to inbreeding depression caused by increased genetic drift (Gigord, Picot, & Shykoff, 1999; Schmitt, 1983). Therefore, fragmented habitats might strongly affect pollinator behavior and plant reproductive success (Ashman & Morgan, 2004; Ashman et al., 2004).

Of the different types of habitats, grasslands are one of ecosystems most severely negatively impacted by human activities. Most areas containing *Zygophyllum xanthoxylum* are ecologically fragile due to human disturbance and rapid desertification. Therefore, the aims of this study were to investigate (1) the role of pollinator and resource limitations during pollination, (2) whether pollinator visits affect the degree of pollen limitation, and (3) whether fragmented habitats affect pollen limitation in *Z. xanthoxylum*. In addition, the reproductive strategies of *Z. xanthoxylum* in these fragmented habitats were characterized.

## 2 METHODS

### 2.1 Study area and fragmentation experiment

Two patches of nutrient‐poor, dry grassland located in the Linze Inland River Basin Comprehensive Research Station in the Gansu Province of China (37°50′–42°40′N, 100°02′–100°21′E) were studied from April 2012 to October 2016.

**Figure 1:**
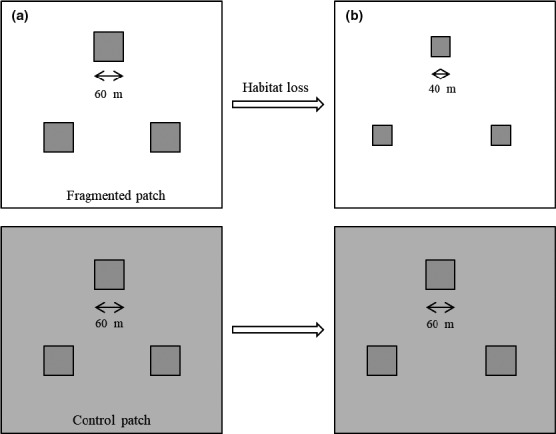
Experimental layout for two studied patches from 2013 to 2016, one is the fragmented patch and the other is the control patch. Three fragmented plots (60 × 60 m) were separated by mown vegetation (white area), as well as the corresponding control patch, which were mirror symmetrically arranged and surrounded by undisturbed vegetation (gray area). (a) Plot size of fragmented patch (60 × 60 m) in 2013; (b) Plot size of fragmented patch (40 × 40 m) in 2016

These two patches of habitat were split into six plots with three plots each in the fragmented and control patches. In the fragmented patch, the plots were divided by areas of vegetation that were frequently mown (five times per year). The corresponding control patch was the same arrangement, and the same plant community as the fragmented patch (Figure 1), but was bordered by undisturbed vegetation. In addition, the average size of the *Z. xanthoxylum* population was similar among the studied plots, and the distance between both patches was approximately 300 m. From 2012 to 2016, the fragmented habitats were disrupted by a decrease in plot size from 3,600 m^2^ to 1,600 m^2^ (Figure 1). In addition to *Z. xanthoxylumm* plants, *Salsola passerine* and *Reaumuria songarica* were also present in the patches. The flowering periods of these species did not overlap with that of *Z. xanthoxylum*.

### 2.2 Plant species

*Zygophyllum xanthoxylum* is primarily found throughout western Inner Mongolia in the western Gansu and Ningxia provinces. Due to the high efficiency of its root system at absorbing water and fixing sand, which make this species resistant to drought and wind, this species plays a critical part in maintaining the stability of the desert ecosystem (Liu, 1987). Unfortunately, populations are fragmented and isolated and have reduced in size over the past 30 years as a result of overgrazing and drought. Moreover, the remaining populations are sparsely distributed and severely damaged, leading to fragmentation, isolation, and reduction in population size. As a result, *Z. xanthoxylum* is now endangered and requires protection in China.

*Zygophyllum xanthoxylum* flowers are relatively small with average petal, sepal, and stamen lengths of 9.12 ± 0.86, 5.71 ± 0.61, and 15.26 ± 1.2 mm (mean ± *SD*), respectively. They have four petals and eight stamens, are typically a yellow hue, and are bisexual.

### 2.3 Flowering, dichogamy, and stigma receptivity

To assess changes in flowering period and anther dehiscence from 2012 to 2016, 20 budding plants were marked randomly in each patch. Over the following 2 weeks, the plants were inspected in the morning and afternoon, and progression through the flowering stages was noted. Ten flowers from each of the marked plants were continuously filmed throughout anthesis. Because *Z. xanthoxylum* can produce protogynous flowers, the receptivity of the stigmas was evaluated in the field using the Peroxtesmo Ko method in 2013 (Dafni & Maues, 1998). To confirm the resulting qualitative findings, virgin stigmas were hand‐pollinated at different stages of flower development, and the pollinated stigmas were then collected, brought back to the lab, and checked for pollen tubes. Over a 12 hr period, the pollen grains were quantified by stereo zoom microscopy and were considered germinated if the pollen tubes were longer than the diameter of the pollen grain was wide (Kearns & Inouye, 1993).

### 2.4 Pollen limitation

In order to assess whether limited pollen availability affected reproductive success, hand pollination was performed on the flowering days. The impact of limited pollen on the seed set, which was measured as number of seeds per flower, was evaluated using three treatments: (1) pollen addition (PA), where flowers were hand‐pollinated by transferring pollen from the center of a flowering inflorescence by hand when flowers had opened; (2) control (C), where the flowers were from manipulated (PA) plants; and (3) procedural control (CC), where additional nonmanipulated individual flowers were selected and left untreated. Importantly, resources may be reallocated between flowers on the same plant, especially when additional pollen has been applied to one flower or inflorescence (Gómez, Abdelaziz, Lorite, Munõz‐Pajares, & Perfectti, 2010). To avoid related confounding effects, PC flowers were evaluated to identify any possible changes in resource allocation following pollen supplementation (Wesselingh, 2007). In addition, hand pollination was performed by saturating the stigma with fresh pollen from a different plant located a minimum of 10 m away.

The pollen supplementation experiment was performed in both the fragmented and control patches. In each patch, 12 healthy plants at the same stage of flowering were randomly labeled. Flower buds on these plants were selected as experimental flowers, where the same number of inflorescences was sampled from each plant. Eight flowers were labeled from the center of eight plants, and then, outcross pollen was hand‐pollinated from four lower flowers for the PA treatment when the flowers had opened. The four upper flowers were left untreated in the same plant as the PA treated flowers for the C treatment. For the four plants remaining from the original 12, four flowers from each plant were CC treated, where each was labeled from the central part of inflorescence. In total, 1,152 flowers were tagged and collected across the two habitats, where there were 6 plots per habitat, 12 plants per plot, one inflorescence per plant, and eight flowers in each inflorescence.

Pollen limitation in this study was estimated based on seed set according to Larson and Barrett (2000):

$${\text{PL}}_{C}=1-\left({\text{RS}}_{C}/{\text{RS}}_{\mathrm{PA}}\right)$$

where RS_C_ and RS_PA_ are the seed sets from the C and PA treatment groups, respectively. Positive values indicate higher reproductive success in the PA treatment group than the C treatment group and, thus, pollen limitation. By contrast, zero or negative values suggest there is no pollen limitation (Fernández, Bosch, Ariza, & Gómez, 2012).

### 2.5 Resource limitation

To evaluate the effect of limiting resources, the same 12 flowering plants in the pollen limitation experiments, six each in the control and fragmented patches, were marked. Three plants in each patch were categorized into control and hand‐pollinated groups, which were further split into three treatment groups based on resource availability: (1) control, where flowers experienced native resources; (2) resource reduction, where 50% of the leaves were removed (and failed to regrow) from the labeled plant prior to flowering inflorescences; and (3) resource addition, where a fertilizer containing liquid nitrogen, phosphorus, and potassium (NPK, 9:2:6) was applied monthly (1% v:v dilution, 20 ml/plant) to the plants during flowering season. The overall aim of these treatments was to estimate the relative impact of hand pollination and resource limitation on flower number and seed set. Therefore, the proportion of flowers on the marked plants was counted throughout video filming. At the end of the fruiting season, the numbers of flowers, ovules per flower, and seeds produced by the C, RR, and RA treatment plants were quantified in the laboratory (Rowan, Jesson, & Burd, 2008).

### 2.6 Pollinator observations

To determine whether there is a relationship between the PL_C_ index and the frequency of visits by pollinators, the two study patches were surveyed for pollinators and the identity and number of visitors from May to July was determined. Overall, approximately 100 flowers were tagged and followed each day between 07:00 and 19:00 hr, where any pollinators gathering nectar and pollen were noted and a DAT‐recorder was used to measure the pollinator visit duration. The visitation frequency to flowers was recorded and calculated according to the following equations (Goverde, Schweizer, Baur, & Erhardt, 2002):

$$\text{Visitation frequency}=\frac{\text{Number of visits}}{\text{Number of flowers}\cdot \text{observation time}}$$

Furthermore, insect nets were used to catch the pollinators, which were then brought back to the laboratory and assessed for the presence or absence of pollen grains on their bodies using a stereomicroscope.

### 2.7 Breeding system

To characterize the breeding system of *Z. xanthoxylum*, 300 flowers at the closed bud stage were marked and assigned to one of the following six treatment groups in each patch: (1) control (natural pollination); (2) manual self‐pollination, where self‐pollen was applied to flowers by hand and then the flowers were bagged to protect against pollination by insects and the wind; (3) spontaneous cross‐pollination, where flowers were emasculated at the bud stage and open‐pollinated; (4) manual cross‐pollination, where the stigma of the emasculated flowers was hand‐pollinated with pollen from different flowers, and the flowers were bagged; (5) emasculation and netting, where the stamens were removed prior to the release of pollen and the flowers were covered in a fine mesh (1 mm^2^) to prevent visits by insects; and (6) emasculation and bagging. At the end of the experiment in October, the number of seeds in the resulting fruit was quantified in the laboratory for each treatment cohort. In addition, the self‐compatibility index (SCI) was calculated according to the following equations (Zapata & Arroyo, 1978):

$$\text{Self‐compatibility index}=\frac{\text{The seed set of manual self‐pollination}}{\text{The seed set of manual cross‐pollination}}$$

where SCI values of ≤0.2 and >0.2 indicate self‐incompatibility and self‐compatibility, respectively.

### 2.8 Data analyses

To determine whether there were differences in seed set size between the pollen limitation treatment groups, repeated‐measures tests using treatment as the within subject factor were performed.

The effect of different treatments on visitation frequency, flower number, and seed set was evaluated using one‐way ANOVA. Statistical significance was assessed using the statistical software package SPSS 19.0 for Windows (SPSS Inc. Chicago, IL, USA).

## 3 RESULTS

### 3.1 Flowering and protogyny of flowers

**Figure 2:**
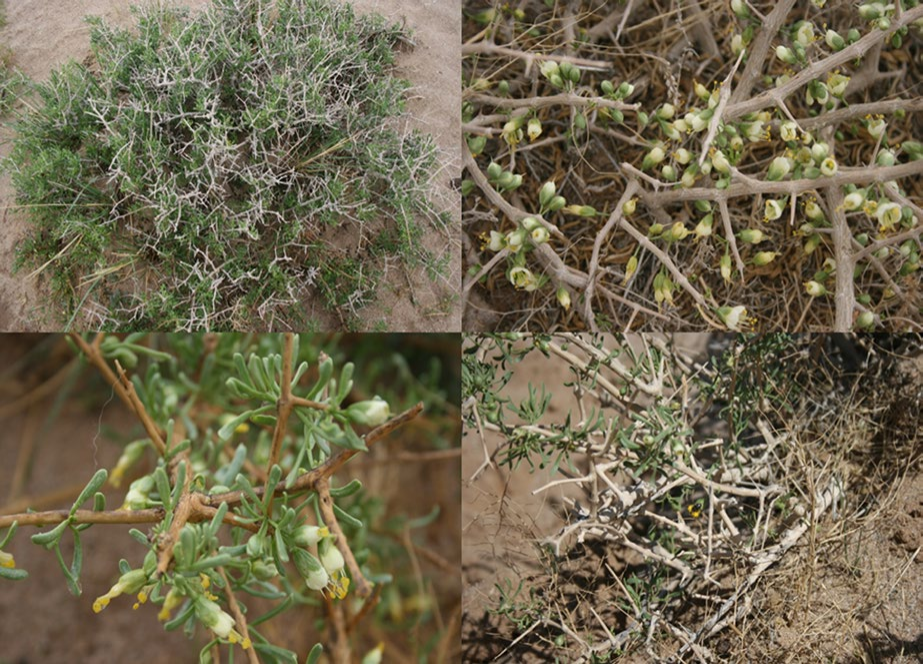
Flowering phenology of *Zygophyllum xanthoxylum* in the study area. It contains flowers in anthesis, completed flowering, and pollinator visited flowers in the fragmented plot

In the study area, flowering of *Z. xanthoxylum* took approximately four days and consisted of female and bisexual stages. No peroxidase activity was noted in the stigma prior to anther dehiscence. At the initial stages of flowering, the anthers were located inside of the corolla. The stigmas remained unreceptive until their corolla had extended during the female stage, which was supported by the presence of pollen tubes after the hand‐pollination experiments. During the bisexual stage, pollen tube growth was restrained throughout the first anther dehiscence. Thus, there was an overall delay in selfing due to difficulty in the stigma accepting pollen and completing selfing because anthers were located below the stigma (Figure 2). In the middle stage of anthesis, this distance between the anthers and stigma was reduced due to filament growth.

### 3.2 Pollen limitation

**Figure 3:**
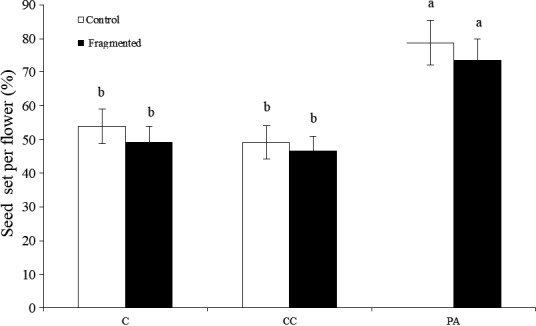
The mean seed set of *Zygophyllum xanthoxylum* under pollen limitation treatments. Vertical bars denote standard error. C, control; CC, procedural control; PA, pollen addition

Pollen limitation was found to be stronger in the fragmented patch (PL_C_ = 0.342 ± 0.036) than in the control patch (PL_C_ = 0.306 ± 0.034). In the control, there were no significant differences observed between the C and CC flower seed sets, where the seed sets were 55.2 ± 5.2% and 49.6 ± 5.1%, respectively. In addition, the average number of ovules per flower was 14.7 ± 3.2. The reproductive output of the procedural control was similar to the control, suggesting adding pollen to separate flowers on the same plant did not divert resources from the accompanying flowers. The PA treatment seed set was 79.6 ± 7.1%, which was significantly larger than seed sets from flowers in the C treatment group (*p* < .05), indicating adding pollen leads to a larger seed set (Figure 3).

In the fragmented patches, the seed sets averaged 50.9 ± 4.7% of the control, 47.3 ± 4.5% of the procedural control, and 77.3 ± 6.5% of the added pollen groups (Figure 3). In addition, pollen supplementation significantly increased the added pollen seed set size compared to the control (*p* < .05). Based on PL indices, pollen limitation in the fragmented patch was more severe than in the control.

### 3.3 Resource limitation

**Figure 4:**
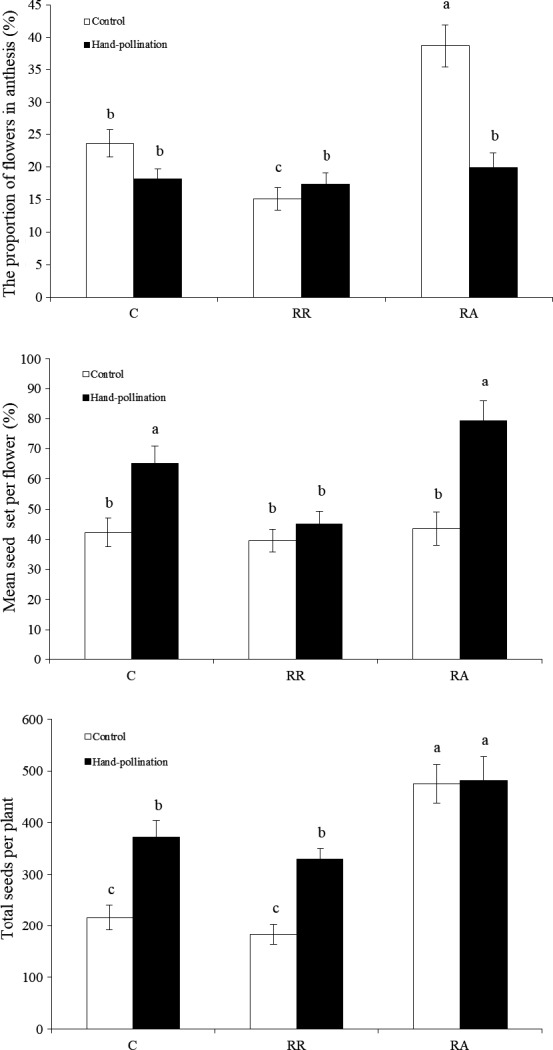
The proportion of flowers in anthesis, the mean seed set per flower and total seeds per plant of *Z. xanthoxylum* under resource limitation treatments. Vertical bars denote standard error. C, control; RR, resource reduction; RA, resource addition

The proportion of flowers in anthesis differed significantly between the open pollination control and the control (23.6 ± 2.1%), resource reduction (15.1 ± 1.7%), and resource addition treatments (38.7 ± 3.2%; *p* < .05). It was found adding resources primarily increased the proportion of flowers in anthesis. In addition, the average size of the seed set in the resource reduced group was only 39.5 ± 3.7%, which was less than the 42.3 ± 4.8% noted in the control and 43.5 ± 5.6% in the resource addition treatment groups (Figure 4). The seed set in the resource addition group was not significantly different from the control treatment (*p* > .05; Figure 4).

Following hand pollination, the mean seed set differed significantly between the control and resource addition groups with 65.2 ± 5.8% and 79.3 ± 6.7%, respectively (*p* < .05). However, the proportion of flowers in anthesis was similar between these groups (*p* > .05; Figure 4). These results indicate hand pollination boosted the seed set size per flower, but had no effect on the proportion of flowers. Furthermore, addition of resources had a slight effect on the total number of seeds per plant (*p* > .05; Figure 4).

### 3.4 Pollinators and visitation frequency

**Figure 5:**
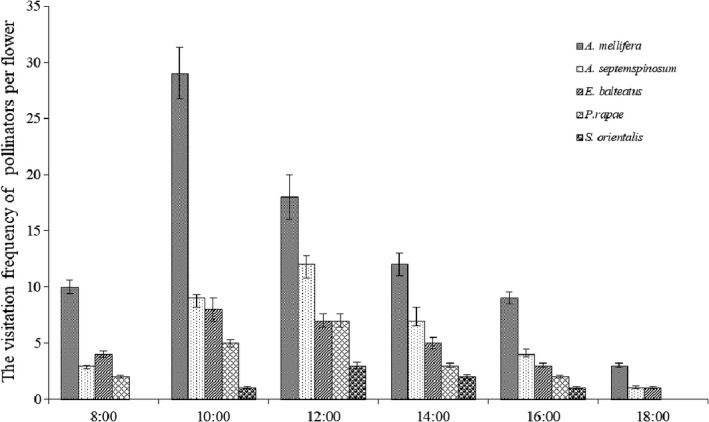
Frequency of pollinators visits to flowers of *Zygophyllum xanthoxylum*

In total, 329 individual pollinator visits were recorded in the study patches. Of these, 203 were *Apis mellifera,* 67 were Hymenoptera, 39 were Lepidoptera, and 17 were Diptera*; A. mellifera* was the most frequent pollinator with significantly more visits than any other pollinator (*p* < .05). *Anthidium septemspinosum* Lepelteier, *Episyrphus balteatus* De Geer, *Pieris rapae* (Linnaeus), and *Serica orientalis* Motschulsky had the highest visiting frequencies. In addition, *A. mellifera* activity primarily coincided with the opening of flowers and release of pollen that occurred between 08:00 and 15:00 (Figure 5). Overall, our results indicate that *A. mellifera* were visiting a higher number of inflorescences and, thus, increasing the efficiency of near‐neighbor pollination.

The mean *V*_f_ of *A. mellifera* differed significantly between the control and fragmented patch at 5.20 ± 0.5% and 3.74 ± 0.4%, respectively. In addition, the PL_C_ index differed significantly between these two cohorts (*p* < .05; Table 1).

**Table 1:** Relationships PL_C_ index (pollen limitation index for seed set) and visitation frequency of *Zygophyllum xanthoxylum*

No.	Plot size	Visitation frequency (visits/(flower hr))	PL_C_ index
Plot 1	23	3.91 ± 0.4^b^	0.34 ± 0.04^a^
Plot 2	16	3.51 ± 0.4^b^	0.39 ± 0.04^a^
Plot 3	18	3.75 ± 0.4^b^	0.36 ± 0.03^a^
Plot 4	20	5.19 ± 0.5^a^	0.31 ± 0.03^b^
Plot 5	19	5.08 ± 0.4^a^	0.33 ± 0.03^b^
Plot 6	17	5.36 ± 0.5^a^	0.28 ± 0.02^b^

Plot size refers to the number of flowering plants in the plot (Plot 1, Plot 2, and Plot 3 are in the fragmented patch, and Plot 4, Plot 5, and Plot 6 are in the control patch), and (*V*_f_) is visits (flower/hr). Different superscript letters indicate significant differences at *p* < .05.

### 3.5 Breeding system

The size of the seed set from each pollination treatment group is shown in Table 2. In the control, the SCI was 0.27 with 21.0% and 76.9% of the seed set under manual self‐pollination and manual cross‐pollination, respectively. These findings indicate the self‐compatibility of *Z. xanthoxylum*. Based on experiments with emasculation and bagging, it was found this species could undergo apomixis, although the seed set was only 3.5 ± 0.7% in the fragmented and 3.1 ± 0.5% in the control.

**Table 2:** Seed set of *Zygophyllum xanthoxylum* in different treatments

Treatment	Seed set (%)	
	Control	Fragmented
Control	52.1 ± 4.7	43.9 ± 3.8
Manual self‐pollination	21.0 ± 2.5	18.6 ± 2.2
Spontaneous cross‐pollination	42.3 ± 3.8	33.6 ± 3.1
Manual cross‐pollination	76.9 ± 7.1	72.3 ± 6.2
Emasculated and netting	13.9 ± 1.5	15.2 ± 1.6
Emasculation and bags	3.5 ± 0.7	3.1 ± 0.5

When using natural pollination, the control seed set was larger than the fragmented set, although this difference was not statistically significant (*p* > .05). In addition, the seed set in the manually cross‐pollinated plants was significantly larger than the control (*p* < .05), suggesting outcrossing successfully enhanced pollination efficiency. The seed set in the group that underwent emasculation and netting was only 13.9 ± 1.5% in the control and 15.2 ± 1.6% in the fragmented patches. In addition, the seed sets in the emasculated and open‐pollinated flowers were significantly larger than the emasculated in the control and fragmented plots (*p* < .05). These outcomes indicate that pollination by insects is critical in the outcrossing system.

## 4 DISCUSSION

### 4.1 Relationship between pollen and resource limitation

The addition of pollen was found to significantly boost the seed set per flower, which is pollen limited in this species. Although resource reduction limited the number of flowers among the remaining flowers, and the seed set produced by the resource‐limited flowers was smaller than the control. Moreover, even after resource addition, the seed set was larger than the control, but there were no significant differences found when comparing the treatments. Evidence from several previous studies suggests that pollen supplementation increases the seed set size per flower due to the requirement of this species for limited pollen (Campbell, 1993; Gómez et al., 2010; Yang et al., 2005). In this study, it was found that supplementing resources had a slight effect on seed set, but the seed set significantly increased when both pollen and resources were added during the flowering season. We concluded that limiting pollen, rather than resources, was responsible for the smaller seed set per flower observed in this species.

Recent reviews have reported that for a plant, many flowers are provided with pollen for the entirety of the plant's lifetime, while in other plants, only a portion of flowers are supplemented with pollen (Knight, Steet, & Ashman, 2006; Ryan & David, 2013). Many studies have also indicated there may be a reallocation of resources by plants across flowers or time (Wesselingh, 2007; Zimmerman & Pyke, 1988). For example, when additional pollen is applied to only one flower or inflorescence on a plant, the resources acquired by that plant may be shifted away from lesser pollinated flowers to the more highly pollinated to support a larger seed set and better seed production. To avoid potential confounding effects, multiple controls were used, including a control in the manipulated plants and a procedural control from those left untouched (Gómez et al., 2010). It was observed that, compared to the control, the procedural control had a lower reproductive output, indicating the addition of pollen to some flowers failed to result in a diversion of resources from neighboring flowers in *Z. xanthoxylum*.

### 4.2 Pollen limitation and pollinators

Pollen limitation may be a result of both limited pollen quantity and quality (Andrea, Julieta, & Andrea, 2008). The importance of quality limitation has rarely been recognized and its magnitude infrequently quantified (Aizen & Harder, 2007). Previous studies have reported that pollen quantity limitation is associated with both pollinator frequency and effectiveness (Ashman et al., 2004). Along these lines, our work also revealed a negative relationship between the frequency of pollinator visits and the PL_C_ index in this species. In addition, empirical evidence indicates that habitat fragmentation can influence pollinator populations both directly and indirectly and may cause a decline in pollinators (Allen‐Wardell et al., 1998). Our studies have demonstrated that pollinators are more likely to visit a population of flowers with a higher density of flowers; therefore, flower population fragmentation reduced the frequency of visitation by pollinators.

Pollination is the first stage of plant sexual reproduction and is necessary for seed development (Kevan et al., 1990). Pollination by animals is generally co‐adaptive, where plants evolve traits to attract particular pollinators, and, in turn, pollinators then evolve traits that allowed enhanced exploitation of the floral resources provided by specific plant species. Habitat fragmentation can change the foraging patterns and visitation rate of pollinators, further reducing the outcrossing success of plants (Dyck & Matthysen, 1999). In this study, we found *A. mellifera* flew longer total distances and lingered longer in the control patches than in the fragmented patches, indicating more near‐neighbor pollination; this may result in more inbreeding in pollinated plants in the control. In plants pollinated by animals, insufficient pollen deposition is likely a result of pollinator assemblage characteristics, such as pollinator species and abundance (Cosacov, Nattero, & Cocucci, 2008). Pollination success is related to the type of pollinator, as different visitors have different degrees of effectiveness pollinating (Gómez et al., 2010). Recent studies have also noted that pollinator activity and frequency had persistent effects on reproduction success (Revel et al., 2012; Wiemer et al., 2012). When evaluating different pollinators, it was found *A. mellifera* was the most effective pollinator, with more legitimate visits and activity than the other pollinators assessed. Notably, *A. mellifera* have large and hairy bodies, which can easily carry and deposit higher amounts of pollen than other pollinators.

### 4.3 Pollen limitation and reproductive strategy in fragmented habitats

Pollen limitation refers to less potential plant reproduction due to inadequate pollen and is a universal occurrence across angiosperms (Ashman et al., 2004; Knight et al., 2005). In this present study, pollen limitation was an important factor in terms of reproductive success. However, changes to habitats can influence pollinator populations either directly or indirectly and may cause a decline in pollinators (Allen‐Wardell et al., 1998; Cresswell, 1997). In addition, fragmentation of habitats affects the visiting frequency and foraging patterns of pollinators, further reducing plant outcross pollination (Goverde et al., 2002). In this work, the PL_C_ index differed significantly between the control and fragmented patches, indicating habitat fragmentation increases pollen limitation in a desert perennial by modulating the rate of pollinator visitation.

Pollen limitation is a strong force driving reproductive strategy and mating system evolution (Ashman et al., 2004). As a result of habitat loss and fragmentation, there may be a reduction in pollinator diversity and abundance. One evolutionary consequence of limiting pollen was the evolution of self‐fertilization. This is because selfing enhances seed production and, therefore, ensures reproductive success in environments unconducive to pollination (Baker, 1955; Morgan & Wilson, 2005). Moreover, self‐compatible plants have little dependence on pollinators and, hence, are less prone to reproductive loss in fragmented areas (Larson & Barrett, 2000). When self‐pollen is more likely to pollinate the ovary than cross‐pollen, self‐pollination may encourage pollination success and, thus, is a selection advantage. Similar reproductive strategies have demonstrated the importance of assistance by self‐pollination when suitable pollinators are scarce in fragmented habitats (Chen, Zhao, & Zuo, 2015; Nayak & Davidar, 2010). In this work, outcross pollination was found to be the dominant strategy in the breeding system, while self‐pollination assisted and ensured the reproduction of *Z. xanthoxylum*. Based on our results, it was challenging for stigma to accept self‐pollen and complete selfing because the anthers were located below the stigma; this led to delayed selfing. In addition, the delayed selfing and protogyny of *Z. xanthoxylum* increased the probability of outcross pollination. The reproductive strategy has been characterized in *Fritillary meleagris* as protogynous with reproduction primarily through outcrossing, making it strongly dependent on insects for pollination for ensuring reproduction assurance (Hedsträm, 1983). Therefore, these reproductive strategies enhance adaptability to harsh environments.

## CONFLICT OF INTERESTS

All authors declare that we do not have any competing financial interests.

## AUTHOR CONTRIBUTIONS

We thank Urat Desert‐grassland Research Station and Naiman Desertification Research Station for all the help and support during this study. MC and XYZ designed the experiment. MC wrote the manuscript; XYZ provided editorial advice.
